# Hæmoptysis as a Sign of Tubercle—Curability of Consumption

**Published:** 1855-09

**Authors:** 


*Hæmoptysis as a Sign of Tubercle; Curability of Consumption; Effect of Cod-Liver Oil, &c.* (Under the care of Dr. Andrew Clark)

The value of hæmoptysis, as an indication of incurable tubercle and consumption, has been a medical question often debated, and this sign or symptom by itself perhaps a little overrated. The chances of pulmonary hemorrhage are, no doubt, increased by whatever tends to diminish the capacity of the chest, as in persons of different trades—tailors, dressmakers, etc.—with crooked spines. Again, in advanced consumption, with cavities, one is too often called upon to witness total destruction of lung tissue and hæmoptysis. In the present instance, however, we wish to speak of the popular and professional notion of phthisis following hæmoptysis as certain, so to speak, as a shadow its substance, and in such matters of every-day practice as signing a certificate of life insurance, hæmoptysis is considered conclusive evidence against doing so. In a large number of cases where hæmoptysis occurs through life, the tendency is evidently towards tuberculosis; a certain proportion of cases probably go on to consumption, while the residue are cured. If, out of 500 cases, 100 or 200 escape, it becomes, an interesting question, what are the general conditions that lead to a cure?—what, on the other hand, are conditions to facilitate the inroads of consumption? It is instructive to compare the result at a general hospital, like the London Hospital or Guy's, and the result at Brompton. At the latter we find, on inquiry, that hæmoptysis takes a very formidable position indeed in the chapter of symptoms preceding tubercle; we find also that cod-liver oil, given in over-doses or in particular cases, has a very manifest tendency to produce hæmoptysis. Amenorrhœa, also, and heart disease, are often attended by this symptom. Leaving all these, however; out of the calculation, we have had reason to be more hopeful of the curability of consumption.

We have been singularly struck with the importance of this question, and with the practical value of the facts springing out of it, from observing the notes of cases, tables, and general results arrived at from the investigations of Dr. Andrew Clark, at the London Hospital. It appears that Dr. Clark entertains the opinion that phthisis of a limited kind is of much more frequent occurrence, and becomes much more frequently cured, than is ordinarily admitted or supposed. This opinion he conceives to be capable of demonstrative proof in three ways: first, by the results of a large number of post-mortem examinations conducted by himself, which show that in the bodies of patients dying from accident, or non-tubercular disease, obsolescent or healed tubercles are found in a very large per centage of cases; secondly, by showing that out of a given number of persons presenting themselves indiscriminately for relief at the London Hospital, many are found to have had hæmoptysis, and that of these a certain proportion has proceeded to the development of unequivocal phthisis, while another proportion has appeared to terminate in complete recovery from symptoms of pulmonary disease; thirdly, by showing that cases of limited chronic phthisis, proved by the presence of air vesicles and tubercular matter together in the sputum, as we have seen it, do not unfrequently proceed to arrestment of the general symptoms, suspension of the progress of the local pulmonary lesion, and subsequent cure.

There can be no doubt that a series of investigations in this threefold aspect, if allowed to their utmost ramifications, and conducted with minuteness and care, will lead to great practical good. In the meantime, without committing ourselves to any specific opinion upon all the questions mooted, we are desirous of commenting upon the second aspect of Dr. Clark's investigations as one which is eminently practical, both in its relations to the disease itself in hospital wards, to the value of hæmoptysis in its bearings upon the signatures to life assurance, as we have already hinted.

In the second aspect of these investigations, Dr. Clark proceeds upon the opinion, that in all cases when hæmoptysis has occurred to the extent of an ounce in the absence of amenorrhœa, aneurism, heart disease, and ulcerated throat, tubercular matter is really present in the lungs. He holds the same opinion even when the hæmoptysis is to a much less amount, provided it be of frequent occurrence, uncomplicated with marked cough and any acute affection of the lung.

Dr. Clark then proceeds to show that, out of a given number of persons applying *indiscriminately* for relief at the London Hospital, and carefully examined relative to this poison, hæmoptysis will be found to have occurred in a large per centage; and that in these instances the hæmoptysis is followed in a certain number of cases by the induction of phthisis, and in a certain proportion by suspension of the symptoms accompanying it and ultimate recovery, and immunity from pulmonary disease. Having established the fact that hæmoptysis, preceded, accompanied, and followed by pulmonary symptoms, often disappears without the return of it or any signs of pulmonary lesion for years afterwards, and sometimes not at all, Dr. Clark then endeavors, as the object of greatest importance in the inquiry, to determine under what conditions this return to health takes place: to what extent, if at all, they can be superinduced by art; and with what amount of certainty we can predict in a given case, termination in recovery, or in phthisis.

We have not space to enter more fully into these details at present. We have pointed out the broad relations of the subject, and shall satisfy ourselves with abstracts of a case or two as types of those which hæmoptysis has not been followed by confirmed phthisis

J. B———, aged sixty-five, a laborer, short, spare, and bent, presented for dyspepsia, accompanied with depresssion and headache. He was delicate in youth, and considered to be "declined" at the age of three or four and twenty. At that time he had cough and expectoration, and freqently spat blood. He used to have pain in the left side, but particularly under the left scapula and about the shoulder. Never had dyspnœa, and never was confined to bed for any length of time. Was very temperate at that time, ate little, and took care of himself. Continued much in the same way for two or three years; was always much subject to colds, and frequently blistered for them. Afterwards began to be better, exposed himself to all weathers, lost his liability to colds, lived rather irregularly, worked hard, took beer and spirits as occasion offered, and lost all note of his symptoms at about thirty, except one, which was pain or gnawing under the left scapula. When he has a cold now, he suffers from a pain in the chest, and has yellow expectoration. Does not often have cold, and for some years has only suffered from occasional bilious fits and rheumatism.

He has, at present, no distinct cough, and no dyspnœa: has a habit, however, of clearing his throat, and expectorates occasionally in the morning, vitreous-looking gelatinous masses, about the size of peas. The pain under the scapula has always recurred at intervals of from three to six months, and lasted variable periods. Left side of chest is flatter than right, and is found, by placing the hands under the axilla, not to fill so well. There is dullness on the left side, jerking respiration, and increased vocal resonance; no rales; around the dullness the lung sounds preternaturally clear, as also does the whole anterior surface of the right lung; heart sounds healthy; every part of chest thrills with the reverbaration of the patient's voice.

We have room for the notes of only one other case, which is a significant and instructive one, but not occurring at the London Hospital:

Mr. B———, aged nineteen, tall, slim, with fair hair, blue eyes, large pupil, transparent skin, and long incurved nails, was seen, in 1846, when the presented all the symptoms of early phthisis. After a time he got better, and was lost sight of for some months. In 1847 he was seen again, and had hæmoptysis, followed by cough, expectoration, night sweats and wasting. No cavity could be made out, but the expectoration contained patches of air vesicles, which Dr. Clark has preserved. He again got a little better, and was moving about when he met with an accident—a fall—which was followed by the formation of an abscess on the upper and outer part of the thigh. The abscess was opened, discharged and continued to discharge a large quantity of matter. Under this exhausting discharge he was with difficulty supported. He became extremely emaciated, and almost helpless. At the same time a remarkable change was observed in the pulmonary symptoms. They began to abate from the day the abscess was opened, and ultimately disappeared entirely. Gradually he regained flesh and strength, and the pulmonary symptoms did not appear. In 1848 he appeared to have perfectly recovered; but contraction having followed the obliteration of the abscess, he had left his former service and become a railway clerk.

On the 9th of November last, Dr Clark met this patient accidentially at the Crystal Palace; and so great, then, was the alteration in his appearance, that Dr. Clark failed at first to recognize him. He was unusually fat for so young a man—corpulent, in fact, and robust looking. He declared himself to be in perfect health, and to have been so for some years. The pupils were still large, and the nails pink, long, and incurved.

Andral states that only in one instance in which hæmoptysis had occurred to him—and even then the immediate cause of death —had he ever found the substance of the lungs free from tubercles. Louis, we need hardly say, gives an equally fatal tendency in two thousand cases in which he made the inquiry, in later years, after the mode by Dr. Andrew Clark; but in eighty-seven private cases under his care, four in six had hæmoptysis.

Pinel gives a singular case of hæmoptysis in a female, which occurred regularly every month, the woman's monthly period, during forty-two years. It was originally caused by fright, and was always subsequently somewhat increased by strong mental excitement. When suspended for a month or two, the patient invariably suffered from intense headaches. She had unusually before the hœmoptysis a sensation of weight and uneasiness abou t the lumbar region and pelvis; soon followed by chillness, lassitude, oppression at the chest, headache, and ultimately a distinct sensation of stinging or bubbling in the bronchial tubes and trachea; then, finally, sharp cough, and spitting of blood The woman was fifty eight years of age when it stopped; she was stout and plump. What conditions here saved her from getting phthisis, like the patients of Andral or Louis, would be an interesting subject of speculating.

The older observers differ very materially as to the frequency of hemorrhage from the lungs in relation to tubercles. Hæmoptysis may precede tubercle for years, and years, even be almost forgotten, till the patient is reminded of it. The use of cod-liver oil, we believe, has very materially changed the rate of mortality and curability of phthisis of late years. Andral found in those dying of phthisis, in his time, that one in six had never hæmoptysis at all; in two in six the hæmoptysis appeared to mark the development of tubercle as a cause; in the remaining number or the half deaths in Paris from phthisis, it followed rather as a consequence from unequivocal phthisical disease, with diarrhœa and wasting , and a breaking up of the lungs into anfractuous sinuses and cavities.—*London Lancet.*

